# Oxethazaine inhibits esophageal squamous cell carcinoma proliferation and metastasis by targeting aurora kinase A

**DOI:** 10.1038/s41419-022-04642-x

**Published:** 2022-02-25

**Authors:** Zhuo Bao, Ang Li, Xuebo Lu, Zitong Wang, Yin Yu, Wenjie Wu, Lili Zhao, Bo Li, Xiangyu Wu, Kyle Vaughn Laster, Chengjuan Zhang, Yanan Jiang, Zigang Dong, Kangdong Liu

**Affiliations:** 1 Department of Pathophysiology, School of Basic Medical Sciences, Zhengzhou University, Zhengzhou, 450000 Henan China; 2 China-US (Henan) Hormel Cancer Institute, Zhengzhou, 450003 Henan China; 3 The Third Affiliated Hospital of Zhengzhou University, Zhengzhou, 450008 Henan China; 4 The Affiliated Cancer Hospital of Zhengzhou University, Zhengzhou, 450000 Henan China; 5 State Key Laboratory of Esophageal Cancer Prevention and Treatment, Zhengzhou, 450052 Henan China; 6 Basic Medicine Sciences Research Center, Academy of Medical Sciences, Zhengzhou University, Zhengzhou, 450052 Henan China; 7 Henan Provincial Cooperative Innovation Center for Cancer Chemoprevention, Academy of Medical Sciences, Zhengzhou University, Zhengzhou, 450000 Henan China; 8 Cancer Chemoprevention International, Collaboration Laboratory, Zhengzhou, 450000 Henan China

**Keywords:** Cancer prevention, Cell growth

## Abstract

Esophageal squamous cell carcinoma (ESCC), a malignant neoplasm with high incidence, is a severe global public health threat. The current modalities used for treating ESCC include surgery, chemotherapy, and radiotherapy. Although ESCC management and treatment strategies have improved over the last decade, the overall 5-year survival rate remains <20%. Therefore, the identification of novel therapeutic strategies that can increase ESCC patient survival rates is urgently needed. Oxethazaine, an amino-amide anesthetic agent, is mainly prescribed in combination with antacids to relieve esophagitis, dyspepsia, and other gastric disorders. In the present study, we found that oxethazaine inhibited the proliferation and migration of esophageal cancer cells. According to the results of in vitro screening and binding assays, oxethazaine binds directly to AURKA, suppresses AURKA activity, and inhibits the downstream effectors of AURKA. Notably, we found that oxethazaine suppressed tumor growth in three patient-derived esophageal xenograft mouse models and tumor metastasis in vivo. Our findings suggest that oxethazaine can inhibit ESCC proliferation and metastasis in vitro and in vivo by targeting AURKA.

## Introduction

Esophageal cancer is the seventh most common cancer in the world [1]. There are two main subtypes of esophageal cancer, esophageal squamous cell carcinoma (ESCC), and esophageal adenocarcinoma. ESCC is the primary histological subtype of esophageal cancer and accounts for 90% of all cases, with regions of the highest incidence localized in eastern Asia and southern Africa [2, 3]. At present, the main treatment methods for ESCC are surgery, chemotherapy, and radiotherapy; however, various adverse effects limit the use of these therapeutic options [4, 5]. Furthermore, the 5-year survival rate is <20% due to recurrence and the lack of preventative strategies after primary treatment [6]. Therefore, there is an urgent need to identify drugs that can effectively prevent the recurrence and metastasis of esophageal cancer.

The Aurora serine/threonine kinase family is highly conserved in eukaryotes and is comprised of three members, Aurora kinase A, B, and C [7, 8]. Aurora kinase A (AURKA) is located on chromosome 20q13.2 and was found to play an important role in mitosis, including centrosome maturation, mitotic entry, mitotic spindle formation, and cytokinesis [9, 10]. AURKA can regulate tumor development by modulating the cell cycle, activating anti-apoptotic signaling, inducing genomic instability, and promoting tumor invasion and tumor cell migration [11–14]. Moreover, AURKA is expressed significantly higher in most tumor tissues relative to normal tissues. Clinical data show that AURKA is over-expressed and amplified in ESCC. In addition, high expression of AURKA is correlated with shorter overall survival and malignant progression [15–17]. Although AURKA plays an essential role in ESCC development, its specific substrates and underlying molecular mechanisms in ESCC remain uncharacterized.

Oxethazaine is an amino-amide compound that is routinely used as a local anesthetic to relieve dysphagia and pain caused by reflux, chronic gastritis, and duodenal ulcers [18–20]. Oxethazaine presents poor solubility and exhibits slow onset and good long-term efficacy. In addition, oxethazaine has a large margin of safety in intragastrical, subcutaneous, intramuscular, and rectal administration routes [21, 22]. Furthermore, oxethazaine has a peculiar chemical structure (‘double‐anesthetic’) and is one of the few local anesthetics that provide analgesia in an acidic environment [18, 23, 24].

In this study, we screened an FDA-approved drug library and found that oxethazaine had strong inhibitory effects on the growth and metastasis of esophageal squamous carcinoma cells (Fig. S1). Using proteomic and phospho-proteomic experiments, as well as SwissTargetPrediction, we found that oxethazaine targets AURKA. Moreover, oxethazaine suppressed tumor growth and metastasis in vivo.

## Results

### Oxethazaine inhibits ESCC cell proliferation and migration

**Fig. 1:**
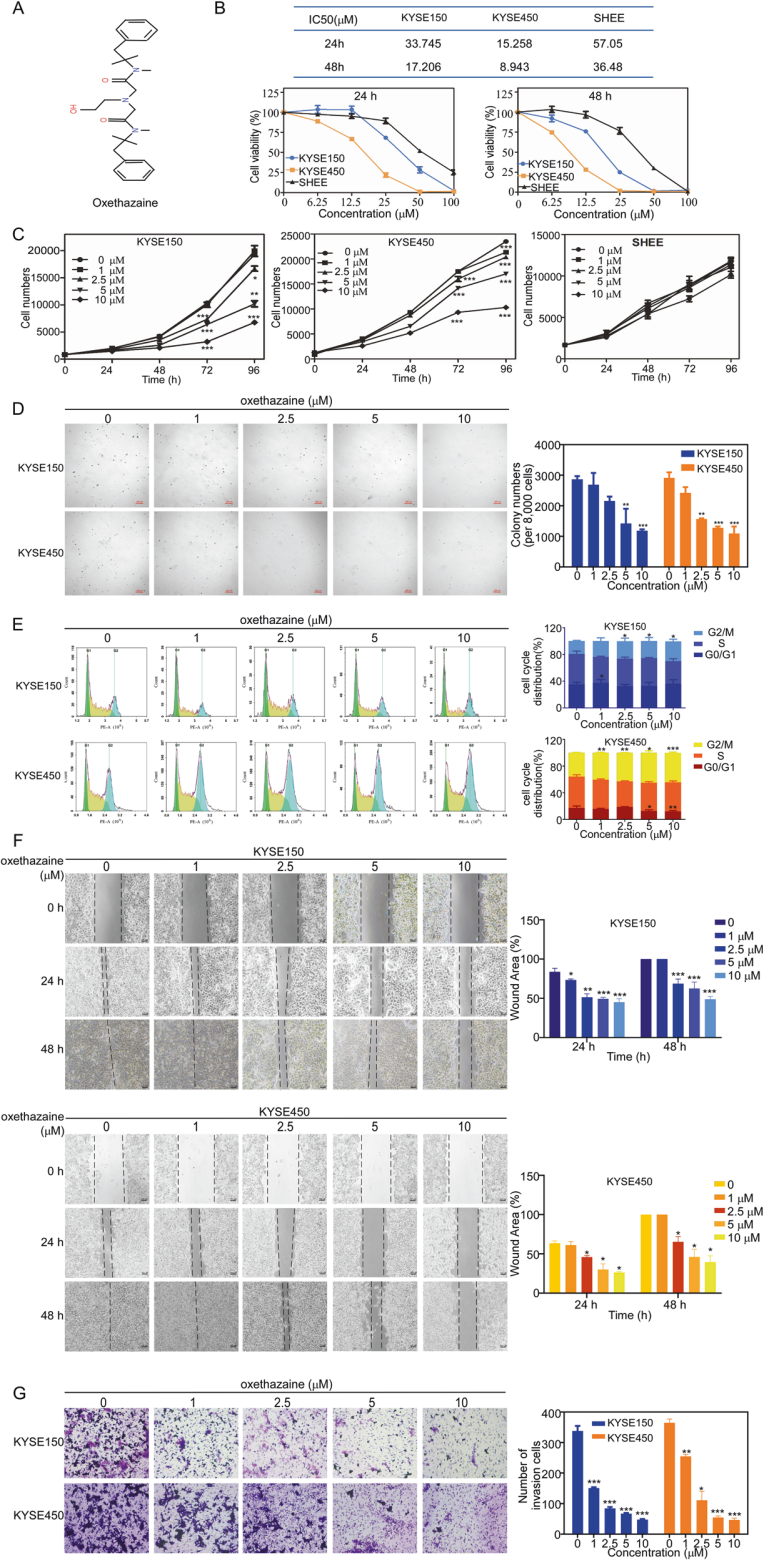
Oxethazaine inhibits the proliferation and migration of ESCC cells. **A** Chemical structure of the oxethazaine. **B** KYSE150 cells, KYSE450 cells, and SHEE cells were seeded in 96-well plates and treated with oxethazaine for 24 h and 48 h. The IC50 value of oxethazaine on ESCC cells and normal esophageal epithelial cells. **C** The cells were treated with various concentrations of oxethazaine (0, 1, 2.5, 5 and 10 µM) and cell numbers were measured at 0, 24, 48, 72 and 96 h using the IN Cell Analyzer 6000 software. **D** Effect of oxethazaine on anchorage-independent growth of ESCC cells. KYSE150 and KYSE450 cells (8 × 10^3^/well) were treated with various concentrations of oxethazaine (0, 1, 2.5, 5 and 10 µM) in an 1.25% Basal Medium Eagle agar matrix containing 10% FBS and cultured for 8 days. Colonies were counted using the IN Cell Analyzer 6000 soft agar program. **E** Cell cycle was analyzed by PI staining and the number of cells in each phase was analyzed by Modfit (*n* = 3). **F** Effect of oxethazaine on cell migration of ESCC cells by wound healing assay. **G** Effect of oxethazaine on cell invasion of ESCC cells with transwell assay. All data are shown as means ± S.D. The asterisks (*, **, ***) indicate statistical significance (*p* < 0.05, *p* < 0.01, *p* < 0.001, respectively).

Oxethazaine is a local anesthetic that relieves dysphagia and pain caused by reflux, chronic gastritis, and duodenal ulcers (Fig. 1A). We treated SHEE (Shantou human embryonic esophageal), KYSE150, and KYSE450 cells with various concentrations of oxethazaine to test cell toxicity and calculated the IC50 values at 24 h and 48 h (Fig. 1B). The IC50 values of SHEE, KYSE150, and KYSE450 cells were 57.05, 33.75, and 15.26 μM at 24 h and 36.48, 17.21, and 8.94 μM at 48 h, respectively. These results indicated that oxethazaine toxicity was lower in the SHEE normal esophageal epithelial cells compared to the KYSE150 and KYSE 450 ESCC cell lines.

To investigate the effects of oxethazaine on ESCC cell growth, we measured the proliferation of KYSE150, KYSE450, and SHEE cells after treatment with various concentrations of oxethazaine (0, 1, 2.5, 5, and 10 μM) using a cell proliferation assay. The results indicated that oxethazaine inhibited ESCC cell proliferation in a dose-dependent manner and had a lower inhibitory effect on SHEE cells (Fig. 1C). The inhibitory effect of oxethazaine on the anchorage-independent growth of ESCC cells was evaluated using a soft agar assay (Fig. 1D), oxethazaine showed strong dose-dependent inhibition of growth. Also, clone formation assays showed that colony formation was suppressed in the oxethazaine-treated groups relative to the control groups (Fig. S2A). Then we performed the flow cytometry to detect the effect of oxethazaine on cell cycle and cell apoptosis in KYSE150 and KYSE450. The results showed that oxethazaine induced a G2/M cell-cycle arrest and apoptosis on ESCC cells (Fig. 1E, Fig. S2B). Furthermore, the results of wound healing assay and transwell assay suggested that oxethazaine reduced the cell migration and invasion compared with control group (Fig. 1F–G, Fig. S2C). Taken together, these results revealed that oxethazaine markedly suppressed cell proliferation and migration in ESCC cells.

### Omic changes of KYSE150 cells after Oxethazaine treatment

**Fig. 2:**
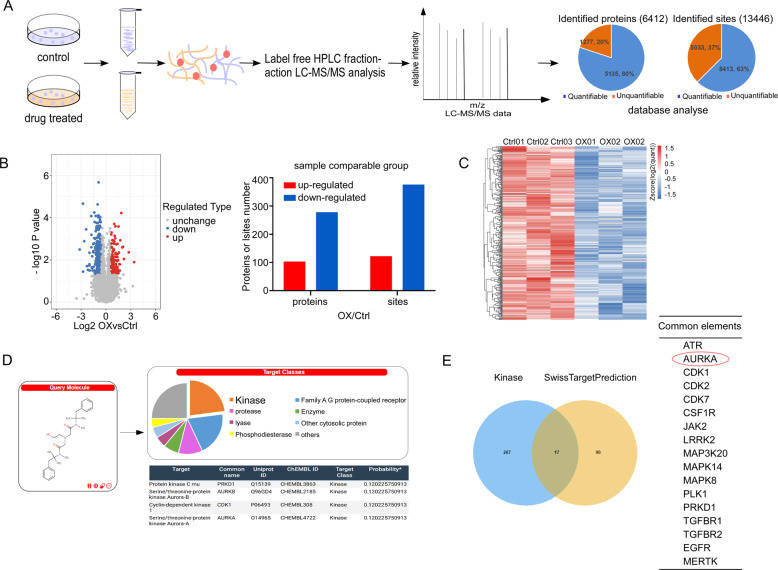
Omic changes of KYSE150 cells after oxethazaine treatment. **A** General workflow of the proteomic and phospho-proteomic experimental strategy used to measure the peptide profiles of KYSE150 cells after 24 h of oxethazaine treatment (10 µM). **B** Volcano plot and statistic histogram shows that 498 sites changed significantly (*p* < 0.05) upon 24 h treatment with 10 µM oxethazaine. Blue and red dots represent down-regulated and up-regulated sites, respectively. **C** Heatmap illustrating differentially expressed proteins between DMSO and oxethazaine (10 µM) groups. **D** SwissTargetPrediction readout generated using the oxethazaine structure. **E** The number of protein predicted by phosphorylation sites (blue, left), the number of protein predicted by SwissTargetPrediction (yellow, right), and the intersection of these two (center).

To further explore the potential mechanism and targets of oxethazaine responsible for its inhibitory effect on ESCC cell proliferation, we conducted proteomic and phospho-proteomic analyses in KYSE150 cells treated with oxethazaine (10 µM) or dimethyl sulfoxide (DMSO) for 24 h. Analysis of the proteomic data identified 6412 proteins, including 5135 proteins with quantitative information. Analysis of the phospho-proteomic data identified 13,446 phosphorylation sites, among which 8413 sites contained quantitative information (Fig. 2A). To evaluate the statistical significance of the results, a strict criterion (*t*-test *p* value < 0.05, FDR < 0.01) was applied in order to filter three biological replicate experiments. Among all quantified phosphorylation sites, we discovered that 498 unique sites were significantly up- or down-regulated (122 and 376 sites, respectively; *p* value < 0.05, fold-change > 1.5, fold-change < 0.67; Fig. 2B). A heatmap illustrating differentially expressed proteins between the DMSO and oxethazaine treatment groups is shown in Fig. 2C. To further investigate how oxethazaine treatment affects protein phosphorylation, we analyzed the differentially phosphorylated residues between the DMSO and oxethazaine treatment groups. Based on the sequence of these phosphorylation sites, we predicted 304 possible upstream regulatory kinases using cytoscape.

We next queried the SwissTargetPrediction web tool using the chemical structure of oxethazaine to predict its potential targets. The results of this analysis identified 107 potential targets of oxethazaine (Fig. 2D). A Venn diagram was then constructed to identify the proteins shared between the predicted upstream kinases and oxethazaine drug targets. The results indicated that AURKA was grouped within the intersecting subset of these datasets (Fig. 2E and Fig. S3).

### Oxethazaine directly binds to AURKA

**Fig. 3:**
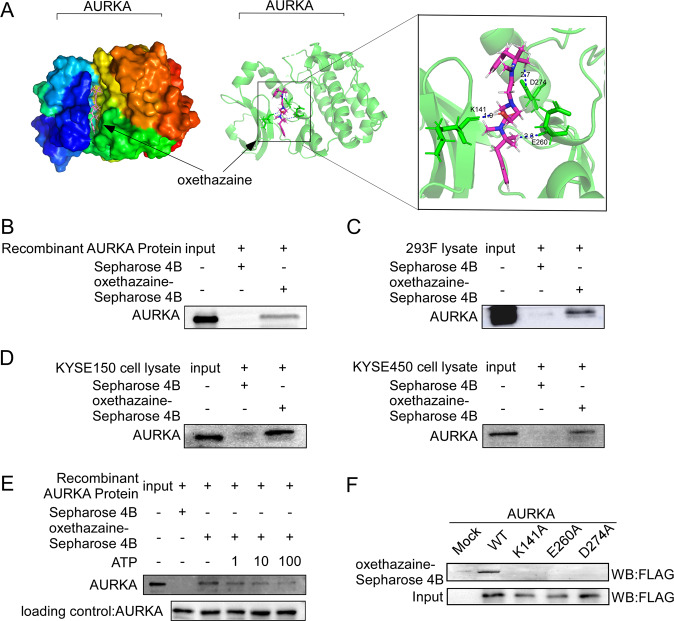
Oxethazaine directly binds to AURKA. **A** Model of oxethazaine binding with AURKA at the K141, E260, and D274 residues and the Ligand Interaction Diagram (LID) of the binding (right panel). **B**–**D** Oxethazaine directly binds to AURKA. The recombinant proteins or cell lysates of 293 F, KYSE150, and KYSE450 cell lines were incubated with Oxethazaine-conjugated Sepharose 4B beads or with Sepharose 4B beads alone. The results were analyzed by Western blotting. **E** The specificity of the binding of oxethazaine to active AURKA in the presence of ATP was evaluated. **F** Cells ectopically expressing AURKA (WT, mutant K141A, E260A, or D274A) were incubated with oxethazaine-conjugated Sepharose 4B beads or with Sepharose 4B beads alone. The results were analyzed by Western blotting.

To determine whether oxethazaine interacts with the AURKA protein, we utilized the Schrödinger Suite 2018 software to conduct computational molecular docking experiments. The results suggested that oxethazaine is able to associate with AURKA at residues K141, E260, and D274 (Fig. 3A). To validate the binding between oxethazaine and AURKA predicted by the in silico analyses, we performed pull-down assays using oxethazaine-conjugated Sepharose 4B beads (or Sepharose 4B beads alone as a negative control) and recombinant AURKA protein. In addition, we performed pull-down assays using cell lysates derived from KYSE150, KYSE450, and the 293 F cell line transfected with AURKA. The results showed that oxethazaine directly binds to the AURKA protein (Fig. 3B–D). Next, we performed an ATP competition assay to verify whether the binding of oxethazaine with AURKA is ATP-competitive. Indeed, the results indicated that oxethazaine competed for binding with ATP at the ATP-biding domain of AURKA (Fig. 3E). The preceding computational docking results suggested that residues K141, E260, D274 of the AURKA protein might be essential for oxethazaine binding. To verify the importance of these residues, we constructed AURKA mutants (K141A, E260A, D274A) and ectopically expressed these constructs in 293 F cells. Pull-down assays using cell lysate derived from 293 F cells expressing either wild-type or mutant AURKA protein and oxethazaine-conjugated Sepharose 4B beads revealed that the binding affinity between the AURKA mutants and oxethazaine was reduced (Fig. 3F), suggesting that these sites are important for binding.

### Oxethazaine could inhibit the kinase activity of AURKA

**Fig. 4:**
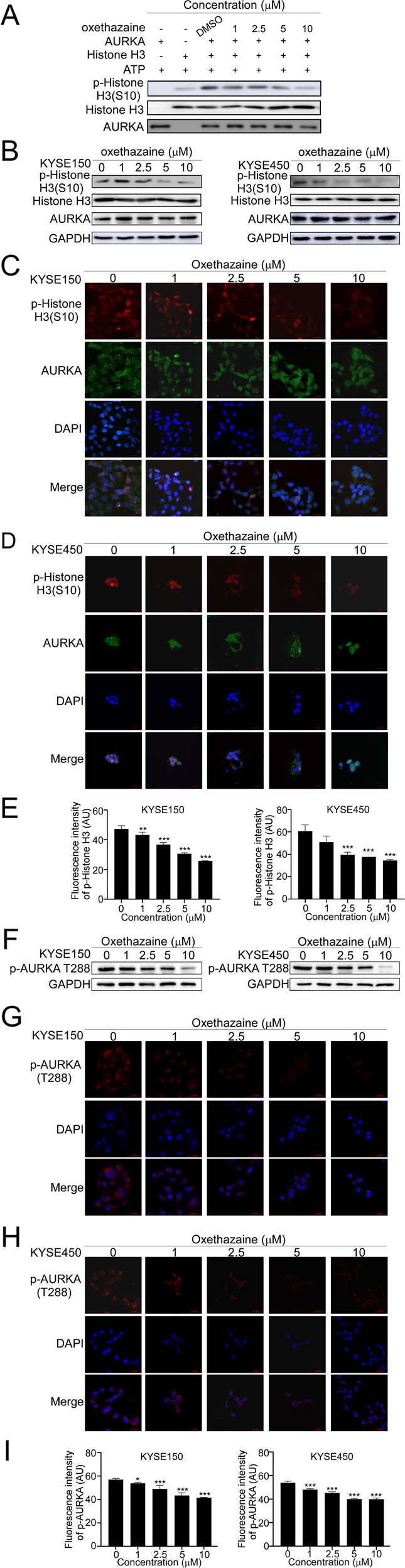
Oxethazaine can inhibit AURKA kinase activity. **A** Oxethazaine suppresses AURKA kinase activity in a dose-dependent manner. **B** Western blotting showing the expression levels of p-Histone H3 (S10), Histone H3, and AURKA after treatment of various concentrations of oxethazaine. **C**, **D** Immunofluorescence of p-Histone H3 and AURKA in KYSE150 and KYSE450 cells treated with oxethazaine for 24 h was photographed using the IN Cell Analyzer 6000. **E** The quantitative analyses of fluorescence intensity in KYSE150 and KYSE450. **F** Western blotting showing the expression levels of p-AURKA (T288) after treatment of various concentrations of oxethazaine. **G**–**H** Immunofluorescence of p-AURKA in KYSE150 and KYSE450 cells treated with oxethazaine for 24 h was photographed using the IN Cell Analyzer 6000. **I** The quantitative analyses of fluorescence intensity in KYSE150 and KYSE450. All data are shown as means ± S.D. The asterisks (*, **, ***) indicate statistical significance (*p* < 0.05, *p* < 0.01, *p* < 0.001, respectively).

According to the prior studies, AURKA physically interacts with the histone H3 tail and effectively phosphorylates Ser10 both in vitro and in vivo. Furthermore, the phosphorylation of histone H3 is a functionally crucial event for the onset of mitosis [25, 26]. Next, we performed in vitro kinase assays with an active recombinant AURKA protein to examine whether oxethazaine affected AURKA activity. Activated recombinant AURKA kinase protein was mixed with human recombinant histone H3 in the presence of various concentrations of oxethazaine to assess whether AURKA kinase activity is inhibited by its presence. The results showed that histone H3 (S10) phosphorylation was reduced upon addition of oxethazaine, indicating that oxethazaine could inhibit the activity of AURKA (Fig. 4A). Western blotting analysis indicated that oxethazaine reduced the level of p-Histone H3 in a dose-dependent manner, but had no effect on the total protein expression levels of histone H3 and AURKA (Fig. 4B). Furthermore, immunofluorescence showed that AURKA and p-Histone H3 overlapped in untreated KYSE150 and KYSE450 ESCC cells; however, treatment with oxethazaine blocked histone H3 phosphorylation, resulting in decreased fluorescence overlap (Fig. 4C–E). Because AURKA kinase activity was increased after phosphorylating at residue T288 in vitro and caused AURKA autophosphorylated [27], we detected the effect of oxethazaine on levels of p-AURKA T288 by Western blotting and immunofluorescence as follow. The results revealed that oxethazaine could reduce the level of p-AURKA T288 (Fig. 4F–I). Collectively, these data indicated that oxethazaine could interact with AURKA and inhibit its kinase assay in ESCC cells.

### AURKA knockout suppresses ESCC cell growth and reduces the sensitivity of ESCC to oxethazaine

**Fig. 5:**
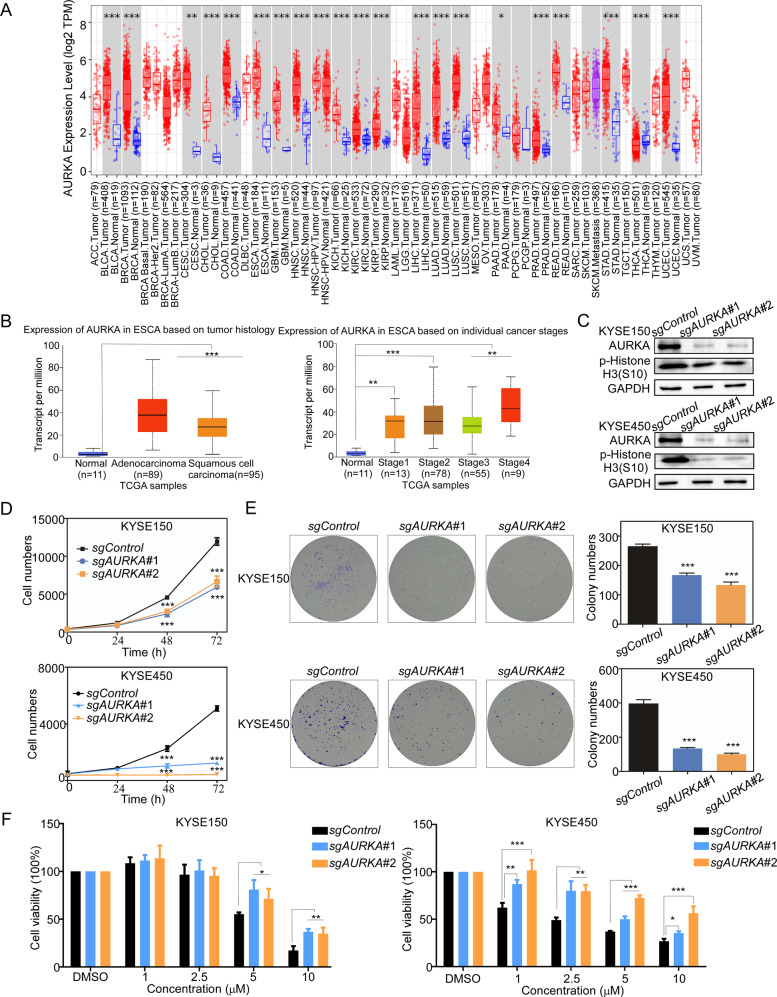
AURKA knockout suppresses ESCC cell growth and reduces the sensitivity of ESCC cells to oxethazaine. **A** The expression of AURKA between tumor and adjacent normal tissues across all TCGA tumors using the TIMER 2.0 website. **B** The expression of AURKA between esophageal tumor tissues and normal tissues. Data are based on TCGA database results from the UALCAN website. **C** CRISPR/Cas9 was used to knockout AURKA in KYSE150 and KYSE450 cells. Knock-out efficiency in KYSE150 and KYSE450 cell lines was assessed by Western blotting. **D**, **E** Cell proliferation and colony formation assay results of KYSE150 and KYSE450 cells transfected with *sg*AURKA-1, *sg* AURKA-2, or *sg*Control. **F** The inhibitory effect of oxethazaine on AURKA knockout cells was detected by proliferation assay after 72 h. All data are shown as means ± S.D. The asterisks (*, **, ***) indicate a significant decrease (*p* < 0.05, *p* < 0.01, *p* < 0.001, respectively).

To further verify whether AURKA is an effective target in ESCC, we first detected the expression of AURKA transcripts in tumor and tumor adjacent tissue samples using the TCGA database. The results showed that AURKA transcript expression in tumor tissues was significantly higher compared to normal tissues (Fig. 5A, B). To further investigate the role of AURKA in ESCC growth, we first transfected KYSE150 and KYSE450 cells with *sg*Control or *sg*AURKA constructs to knockout (KO) *AURKA gene*. The transfection efficiency in the KYSE150 and KYSE450 cells was measured by Western blotting (Fig. 5C). We then performed a cell proliferation assay using the AURKA KO cells to assess the contribution of AURKA to ESCC cell growth. The results of the proliferation assay indicated that cell proliferation of the AURKA KO cells was significantly reduced relative to the *sg*Control cell lines. Similarly, the colony formation ability of the AURKA KO ESCC cells was inhibited (Fig. 5D, E). We also investigated whether the inhibitory effect of oxethazaine on ESCC cells was dependent upon AURKA expression by performing a proliferation assay using the AURKA KO cells. The results revealed that the inhibitory effect of oxethazaine on the proliferation of AURKA KO ESCC cells was decreased compared to the *sg*Control group (Fig. 5F). These results indicated that AURKA plays an essential role in ESCC cell growth and reduces the sensitivity of ESCC cells to oxethazaine.

### Oxethazaine inhibits ESCC tumor growth and metastasis in vivo

**Fig. 6:**
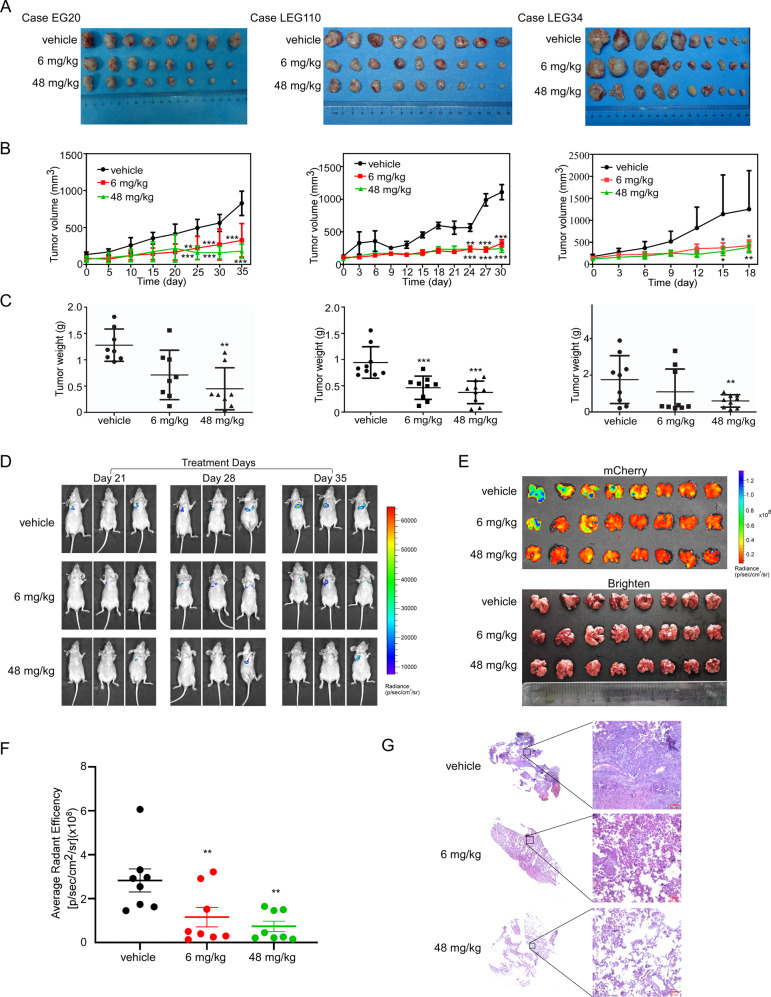
Oxethazaine inhibits ESCC tumor growth and metastasis in vivo. **A** Photographs of tumors for EG20 (*n* = 8), LEG110 (*n* = 9), LEG34 (*n* = 10). **B** Tumor growth curve after oxethazaine treated for EG20 (*n* = 8), LEG110 (*n* = 9), LEG34 (*n* = 10). **C** Tumor weight after oxethazaine treated for EG20 (*n* = 8), LEG110 (*n* = 9), LEG34 (*n* = 10). **D** Representative images for metastasis location of mice in the vehicle and oxethazaine treatment group are shown. **E** Representative images for lung metastasis in the vehicle and oxethazaine treatment group are shown. **F** Effect of oxethazaine treatment on tumor metastasis compared to the vehicle-treated group. The asterisks (**) indicate a significant (*p* < 0.01) decrease in mCherry fluorescent radiant efficiency compared to control. **G** HE staining was used to detect the pathological changes of mice lung.

To investigate the anti-tumor effect of oxethazaine in vivo, we utilized the EG20, LEG110, and LEG34 ESCC PDX-models (Fig. S4A). PDX mice were treated with vehicle (0.9% normal saline) or oxethazaine (6 mg/kg or 48 mg/kg) by oral gavage every day until the average tumor volume of the control group reached 1000 mm^3^. The in vivo experiment results indicated that the tumor volume and weight were significantly decreased in the oxethazaine group compared to the vehicle group; notably, the body weights of the mice were comparable between the vehicle and treatment groups (Fig. 6A–C, Fig. S5A). We also quantified the changes in tumor volume of each tumor across the PDX models. The results illustrate the inhibitory effect of oxethazaine on tumor growth more clearly (Fig. S4B). We then determined the effect of oxethazaine on Ki67, a proliferation marker, using immunohistochemistry. The results showed that Ki67 expression was significantly decreased within the oxethazaine treatment groups (Fig. S4C). We also detected the expression of p-Histone H3 (S10) using immunohistochemistry. The results indicated that p-Histone H3 (S10) expression was similarly suppressed in the oxethazaine treatment group compared to the vehicle-treated group (Fig. S4D). To further verify the effect of oxethazaine on tumor metastasis of esophageal cancer, Luciferase-mCherry-labeled KYSE150 tumor cells were injected into nude mice through tail vein. Compared to the vehicle-treated group, the oxethazaine-treated group exhibited suppression of KYSE150 cell lung metastasis (Fig. 6D–G). Meanwhile, the body weights of the mice were comparable between the vehicle and treatment groups (Fig. S5B). These results suggest that oxethazaine markedly decreased ESCC tumor growth and metastasis in vivo.

## Discussion

Currently, the development and approval of new drugs is a time-consuming and costly endeavor, which may face unexpected clinical side effects and tolerance, therefore, drug repurposing has received more attention [28, 29]. Now there are some non-cancer drugs for cancer prevention or inhibition, such as cardiovascular drugs, non steroidal anti-inflammatory drugs, antipsychotics and antidepressants, antibiotics and antiviral drugs [30–32]. Previous studies of our research group found that antitussive agent cloperastine and 5-hydroxytryptamine 4-receptor partial agonist tegaserod maleate have a good inhibitory effect on the proliferation of ESCC and can effectively prevent the recurrence of ESCC [33, 34]. These studies show that drug repurposing is of great significance for the development of cancer prevention drugs.

Therefore, we screened FDA-approved drugs for compounds that may prevent ESCC recurrence. In the present study, we found that oxethazaine, a local anesthetic, was a novel AURKA inhibitor (Figs. 3 and 4) and suppressed the proliferation and migration ability of ESCC cells in vitro (Fig. 1) and patient-derived esophageal tumor growth and tumor metastasis in vivo (Fig. 6). Oxethazaine is a local anesthetic that can provide analgesia at low pH. It is mainly used to relieve pain caused by esophagitis, indigestion, and other stomach disorders [18, 20]. Oxethazaine is safe for intragastric, subcutaneous, intramuscular, and rectal administration; however, the toxicity of intravenous or intrapulmonary injection is high [23]. In our study, we used the intragastric administration route to ensure safety and avoid the danger associated with intravenous administration. Although oxethazaine has long been used in clinical applications for its local anesthetic effects [35, 36], there are only a few reports on its mechanism of action. In particular, there have been no prior studies on oxethazaine and cancer, and its mechanism of action in this context has not been characterized.

AURKA, as an oncogene, is involved in tumor occurrence through various mechanisms, thus providing a potential target for tumor therapy [37–40]. Previous studies have shown that AURKA is over-expressed in ESCC [41], and increased AURKA expression is correlated with the degree of ESCC malignancy. In addition, AURKA over-expression contributes to the emergence of drug resistance to anticancer agents [42, 43]. Thus, AURKA may serve as a potential prevention or therapeutic target. AURKA kinase domain (aa 125-391) consists of the smaller N-terminal lobes, a single α helix(αC) and an additional helix preceding αC which contains a activation loop (A loop) [44]. Lys 141 was loacted in β1 of stranded antiparallel β1-5 sheets which formed the smaller N-terminal lobes. Asp 274 was the first animo acid of a activation loop (A loop; residues 274–297) which was involved in polypeptide substrate binding. Glu260 was located one of the helixes which formed the ATP binding pocket. Our results found that oxethazaine could bind to these three important sites (Lys 141, Glu 260 and Asp 274). These data indicated that oxethazaine interferes both ATP binding to the ATP cavity of AURKA and the substate binding to A loop. Of note, considerable conformational variability was observed in AURKA kinase, as result of regulation of AURAKA kinase [44]. We speculated that there were the conformational changes in the the relative orientation of the N lobe, αC and A loop once oxethazaine bound with AURKA kinase protein, resulting in the change of the position of Arg180, His176, and Arg 255. The side chains of R180, H176, and R255 created the cavity for phosphate ion which could bind with Thr 288. This may be explain that oxethazaine can down-regulate the expression of phos-AURKA T288. Therefore, oxethazaine may be a potent AURKA inhibitor.

In the past decades, several AKIs (AURKA kinase inhibitors) have been identified, which show anti-proliferation activity in some solid tumors and hematological malignancies. Some of them have already been investigated in clinical trials [41]. MLN8237 and ENMD-2076, two AURKA inhibitors, have shown efficacy in clinical trials [45–48]. AKIs have also been reported to be combined with chemotherapy, immunotherapy and other targeted therapies to improve the effect of tumor therapy [40]. Although targeted AKIs have demonstrable effects on tumors, most AKIs have failed clinical or preclinical evaluation due to toxicity, including neutropenia, somnolence, and mucositis [49–51]. Oxethazaine, as a FDA-aproved drug, has already proved itself in clinical trials, therefore, it has good potential for tumor therapy as a new AURKA kinase inhibitor with low toxicity.

Our findings identified oxethazaine as a novel drug with chemopreventive potential beyond the original use and demonstrate that oxethazaine is a potent AURKA inhibitor. Thus, oxethazaine could be useful in the prevention of ESCC progression.

## Materials and methods

### Chemicals and reagents

Oxethazaine was purchased from TargetMol (#T0044, USA) and Toronto Research Chemicals (#O846600, Canada). Primary antibodies against AURKA was purchased from Abcam (ab13824, USA) and Cell Signaling Technology (#14475, USA), primary antibodies against p-AURKA (#3079), p-Histone H3 (#9701), Histone H3 (#9715) were purchased from Cell Signaling Technology (Danvers, MA, USA). DAPI, RNase and PI were purchased from Solarbio Science & Technology Co (Beijing, China).

### Cell lines and cell culture

Shantou human embryonic esophageal (SHEE) cells were obtained from professor Enmin Li of Shantou University. The KYSE150 and KYSE450 ESCC cell lines were purchased from the Chinese Academy of Sciences Cell Bank (Shanghai, China). The cells were cytogenetically tested by STR-Promega and authenticated. Cells were cultured as previously described [52].

### Cell viability assay

KYSE150 (3 × 10^3^ cells per well), KYSE450 (5 × 10^3^ cells per well), and SHEE (3 × 10^3^ cells per well) cells were seeded into 96-well plates. After incubation for 16–18 h, the cells were treated with various concentrations of oxethazaine (0, 6.25, 12.5, 25, 50 and 100 µM) for 24 or 48 h. Cell nuclei were stained with 4′,6-diamidino -2-phenylindole (DAPI) and counted with In Cell Analyzer 6000 (GE Healthcare, USA).

### Cell proliferation assay

KYSE150 (3 × 10^4^ cells per well) and KYSE450 (5 × 10^4^ cells per well) cells were seeded into 96-well plates. After treatment with various concentrations of oxethazaine (0, 1, 2.5, 5, and 10 µM) for 24, 48, 72, and 96 h, the cell nuclei were stained with DAPI and counted using an In Cell Analyzer 6000 (GE Healthcare, USA).

### Anchorage-independent cell growth assay

The anchorage-independent cell growth assay was performed as described previously [52]. Cells were seeded into six-well plates with 8000 cells per well with or without oxethazaine treatment. Colonies were photographed and counted using an In Cell Analyzer 6000 (GE Healthcare, USA).

### Clone formation assay

Cells were seeded at 200–300 cells/well into six-well plates and treated with various concentrations of oxethazaine (0, 1, 2.5, 5, and 10 µM) for 8 days. Cells were stained with 0.1% crystal violet after 4% paraformaldehyde fixation, and then photographed for quantification.

### Cell cycle assay

Cells were seeded into 60 mm dishes (2 × 10^5^ cells per dish). After starved for 24 h, the cells were treated with various concentrations of oxethazaine (0, 1, 2.5, 5, and 10 µM) for 48 h. The cells were fixed with 70% cold ethanol after collected by trypsinization. Then cells were incubated with RNase and stained with PI (50 μg/mL) and analyzed by Flow Cytometer (BD Biosciences, San Jose, CA).

### Apoptosis assay

The apoptosis assay was performed as described previously [52]. Cells were seeded into 60 mm dishes (2 × 10^5^ cells per dish) and incubated with or without oxethazaine treatment. Cells were stained with annexin V (BioLegend, San Diego, CA) and PI, then analyzed by flow cytometry.

### Wound healing assay

KYSE150 and KYSE450 cells were seeded into six-well plates (4 × 10^5^ per well). After reached 100% confluence, cells were scratched with a pipette tip. Then cells were treated with various concentrations of oxethazaine (0, 1, 2.5, 5, and 10 µM) for 24 h and 48 h and taken photographs at the same area. The migration distance was assessed by ImageJ.

### Transwell migration and invasion assay

Tanswell migration and invasion assay were performed as described previously [53]. Cells were seeded into 24-wells-plates (1.5 × 10^4^ per well) with uncoated (for migration) and matrigel-coated (for invasion) Transwell chambers (8 μm, Corning, NY, USA). After incubated with or without oxethazaine, cells were fixed with 4% paraformaldehyde and stained with 0.1% crystal violet. Then photographed and identified by microscopy.

### Mass spectrometry proteomics analysis

After treating KYSE150 cells with 10 μM oxethazaine for 24 h, cells were collected for protein extraction. After trypsin digestion, peptides were desalted by Strata X C18 SPE column (Phenomenex) and vacuum-dried. Peptides were reconstituted in 0.5 M TEAB and processed according to the manufacturer’s protocol for the TMT kit/iTRAQ kit. Next, the tryptic peptides were fractionated by high pH reverse-phase HPLC using a Thermo Betasil C18 column (5 µm particles, 10 mm ID, 250 mm length). The peptides were then subjected to NSI source followed by tandem mass spectrometry (MS/MS) in Q ExactiveTM Plus (Thermo) coupled online to the UPLC. The resulting MS/MS data were processed using the Maxquant search engine (v.1.5.2.8).

### Western blotting assay

Protein concentrations were determined using a BCA Quantification Kit (Solarbio). Total cellular protein extracts were separated by sodium dodecyl sulfate-polyacrylamide gel electrophoresis (SDS-PAGE) and transferred to PVDF membranes in a transfer buffer. The membranes were blocked in 5% nonfat dry milk in 1×TBS for 1.5 h and incubated with primary antibodies overnight at 4 °C. Blots were washed three times in 1× TBS buffer and incubated with secondary antibody for 2 h. Proteins were then detected using ECL regent.

### Computational modeling of AURKA interaction with oxethazaine

The computational docking was performed as described as previously [54]. The AURKA crystal structure (PDB:1MQ4) was obtained from the Protein Data Bank (https://www.rcsb.org/). The oxethazaine chemical structure was prepared for docking using the default parameters of the LigPrep program.

### Pull-down assay

Active recombinant AURKA protein (200 ng) and whole-cell lysates (500 µg) were incubated with oxethazaine-Sepharose 4B (or Sepharose 4B only as a control) beads in reaction buffer. After incubation overnight with rotation at 4 °C, the beads were washed three times with buffer, and bound protein was verified by Western blotting after elution. Buffer composition was used as described previously [54].

### In vitro kinase assay

The active recombinant AURKA protein (100 ng) was mixed with various concentrations of oxethazaine in 10× buffer and incubated at room temperature for 15 min. Next, the inactive histone H3 recombinant protein, ATP, and 1× buffer were added and incubated at 30 °C for 30 min. The reaction was terminated with the addition of 5 μL of protein loading buffer. The mixture was resolved using Western blotting. AURKA activity was detected using a p-histone H3 antibody.

### Immunofluorescence staining

ESCC cells were seeded into 24-well plates with glass slides and treated with various concentrations of oxethazaine (0, 1, 2.5, 5, and 10 µM) for 24 h. After washing with 1×PBS, cells were fixed with 4% paraformaldehyde for 30 min. Permeabilized with 0.5% Triton X-100 for 10 min after washing with 1×PBS. Then the cells were incubated with primary antibodies AURKA, p-Histone H3 or p-AURKA T288 diluted in 3% bovine serum albumin in PBS overnight at 4 °C and with secondary antibody FITC (#F-2761, Invitrogen), Cyanine3 (#A10520, Invitrogen) or Dylight 594 (#A23420, Abbkine) for 2 h, respectively. Finally, cell nuclei were stained with DAPI for 15 min at room temperature. The images were captured by IN Cell Analyzer 6000 and analyzed by Image J.

### CRISPR/Cas9 knockout cell lines

AURKA was deleted in ESCC cells by using the CRISPR/Cas9 system. Targeting nucleotides were designed using http://chopchop.cbu.uib.no/search.php. Oligonucleotides were inserted into Lenti‐CRISPR‐V2 vector. Following the manufacturer’s suggested protocols, viral vectors and packaging vectors were transfected into HEK293T cells by using Jet Primer (ThermoFisher Scientific, Waltham, MA, USA). The transfection process was performed according to a previously described protocol [52]. Transduction efficiency was assessed by Western blotting.

### ESCC PDX mouse model

This study protocol was approved by the Zhengzhou University Institutional Animal Care and Use Committee and the Research Ethics Committee of Zhengzhou University (Zhengzhou, Henan, China). ESCC tissues were obtained from the Linzhou Hospital of Henan Province and written informed consent was obtained from all patients for the use of their tissue samples. The tissue was trimmed to a proper size (0.10–0.12 g) and inoculated into the back neck of female SCID mice aged 6–8 weeks. Once the tumor nodules grew to ~200 mm^3^, the mice were randomly divided into three groups: (1) vehicle; (2) oxethazaine treatment (6 mg/kg); (3) oxethazaine treatment (48 mg/kg). Oxethazaine or vehicle were administered by gavage six times per week. Body weight and tumor volume were measured once or twice per week. When the control tumor mass volume reached ~1000 mm^3^, the mice were anesthetized and the tumors were excised and processed for subsequent analyses.

### Tumor metastasis mouse model

Luciferase-mCherry stable expressed KYSE150 cells were established by transfering p-PGK-Luciferase-mCherry plasmid. The cells were injected into BALB/c nude mice (Vital River, Beijing, China) via tail vein. The mice were randomly divided into three groups: (1) vehicle; (2) oxethazaine treatment (6 mg/kg); (3) oxethazaine treatment (48 mg/kg). The day after cell injection, oxethazaine or vehicle were administered once a day. After 21, 28, and 35 days, the mice were subjected to in vivo imaging to monitor the luciferase fluorescence expression after injecting the D-Luciferin, potassium salt by IVIS^®^ Lumina III In Vivo Imaging System. At 35 days, the mice were euthanized after measuring the luciferase fluorescence of mice, then mCherry fluorescence expression of the lung was measured as described previously [55]. This study protocol was approved by the Zhengzhou University Institutional Animal Care and Use Committee and the Research Ethics Committee of Zhengzhou University (Zhengzhou, Henan, China).

### Immunohistochemistry analysis

The tumor tissues from mice were embedded in paraffin, cut into 4 μm sections, placed onto slides, and subjected to immunohistochemistry. Tissue sections were deparaffinized, hydrated, and processed for antigen retrieval. After incubation with 3% H_2_O_2_ for 5 min to inactivate endogenous peroxidases, the tissues were incubated overnight with primary antibodies at 4 °C. The tissue sections were washed three times with 1× TBST and then incubated with an HRP-IgG secondary antibody at 37 °C for 15 min. After DAB staining, all slides were stained with hematoxylin, dehydrated, and mounted under glass coverslips.

### Statistical analysis

All quantitative results are expressed as mean values ± SD. Significant differences were determined using Student’s *t* test or one‐way analysis of variance. The Statistical Package for the Social Sciences for Windows (IBM, Inc. Armonk, NY, USA) was used to calculate *P* values. Statistical significance was set at *p* < 0.05.

## Supplementary information

Reproducibility checklist

Supplemental figure

## Data Availability

The data supporting the findings of this study can be found in the article, Supplementary information or available from the corresponding author upon reasonable request.
