# Supplementary material for: Oxethazaine inhibits esophageal squamous cell carcinoma proliferation and metastasis by targeting aurora kinase A
Source: Cell Death Dis. 2022 Feb 25;13(2):189. doi: 10.1038/s41419-022-04642-x (PMC8881465; doi:10.1038/s41419-022-04642-x)
Supplement: Supplementary file 2 — Supplemental figure [file 41419_2022_4642_MOESM2_ESM.docx]

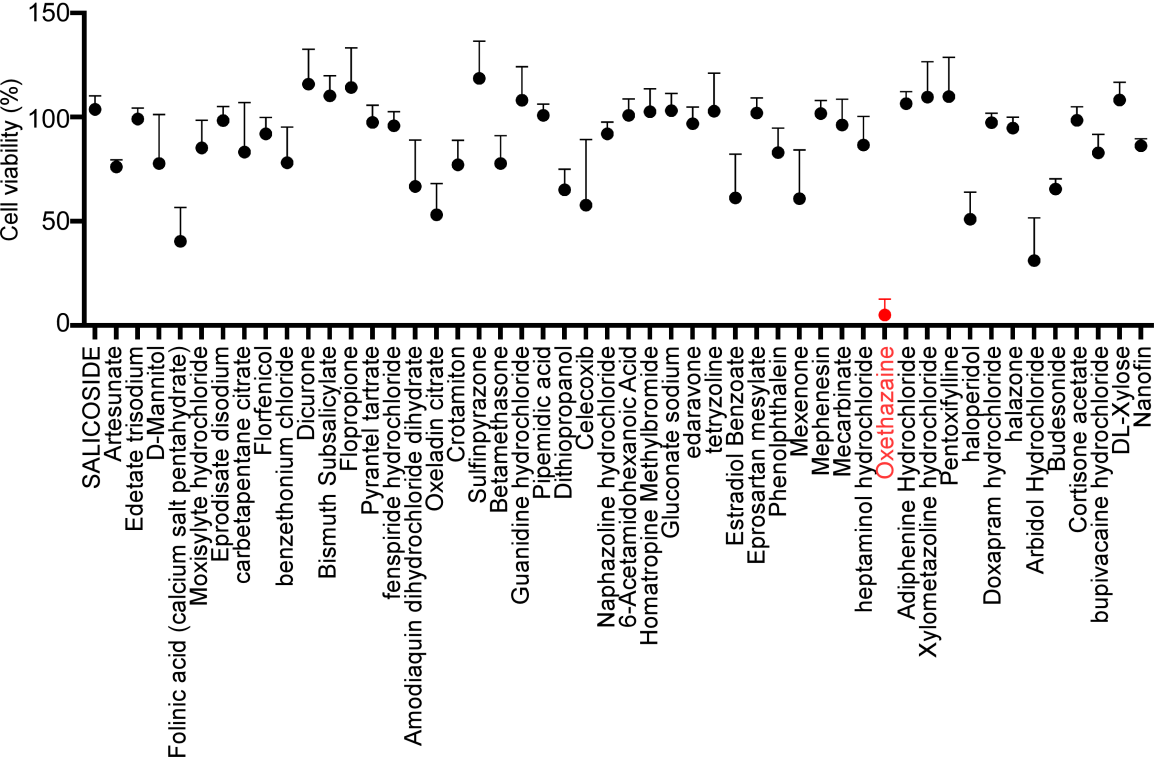


Fig S1. Screening 50 kinds of drugs in FDA-approved library by cytotoxicity assay in KYSE450 cells (n=3).


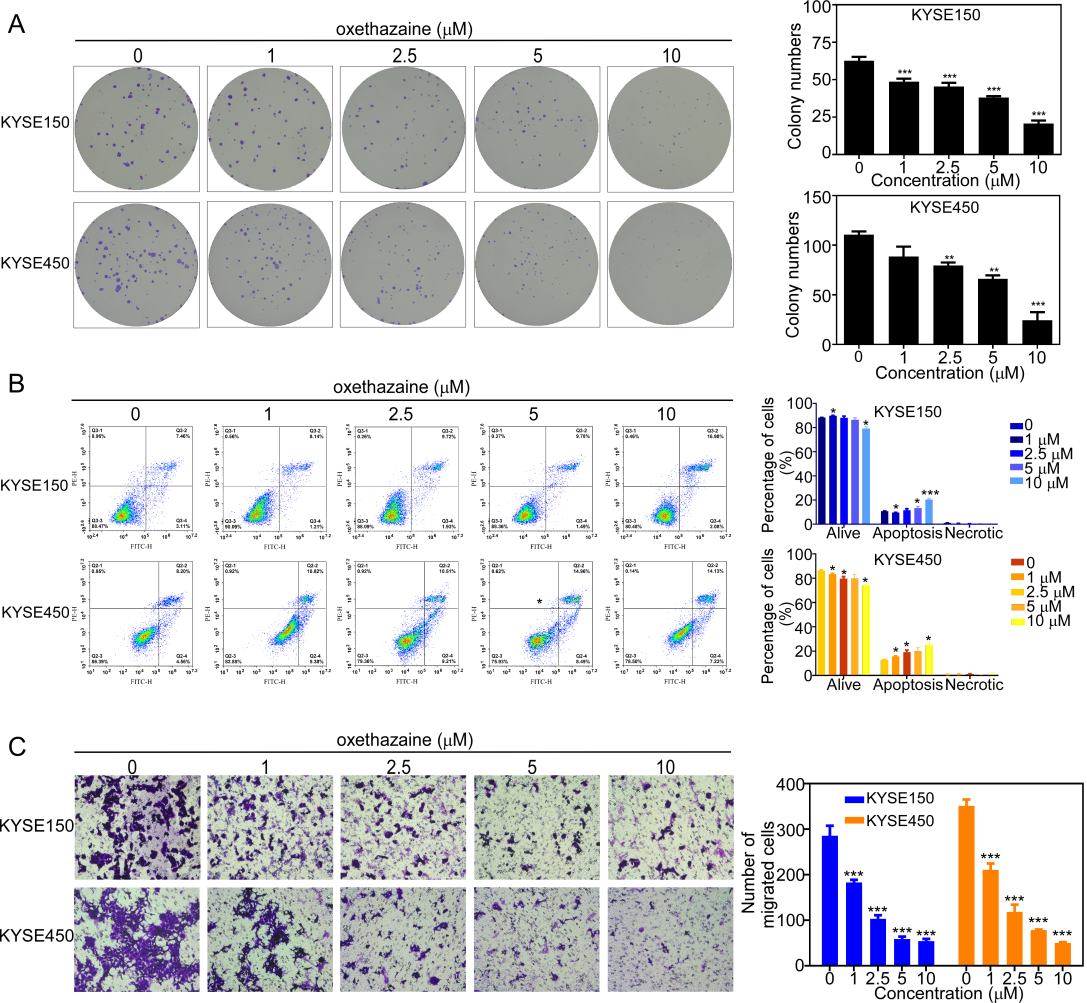


Fig S2. Effect of oxethazaine on anchorage-independent growth，apoptosis and migration of ESCC cell. (A) Effect of oxethazaine on anchorage-independent growth of ESCC cell. KYSE150 and KYSE450 cells (250 cells/well) were treated with various concentrations of oxethazaine (0, 1, 2.5, 5 and 10 µM) in Basal medium containing 10% FBS and cultured for 8 days; the culture medium was changed every 3 days. (B) Effect of oxethazaine on cell apoptosis of ESCC cells. Cells were stained with annexin V and propidium iodide (PI) and apoptosis was determined by Fluorescence Activated Cell Sorting (FACS). (C) Effect of oxethazaine on cell migration of ESCC cells with transwell assay.


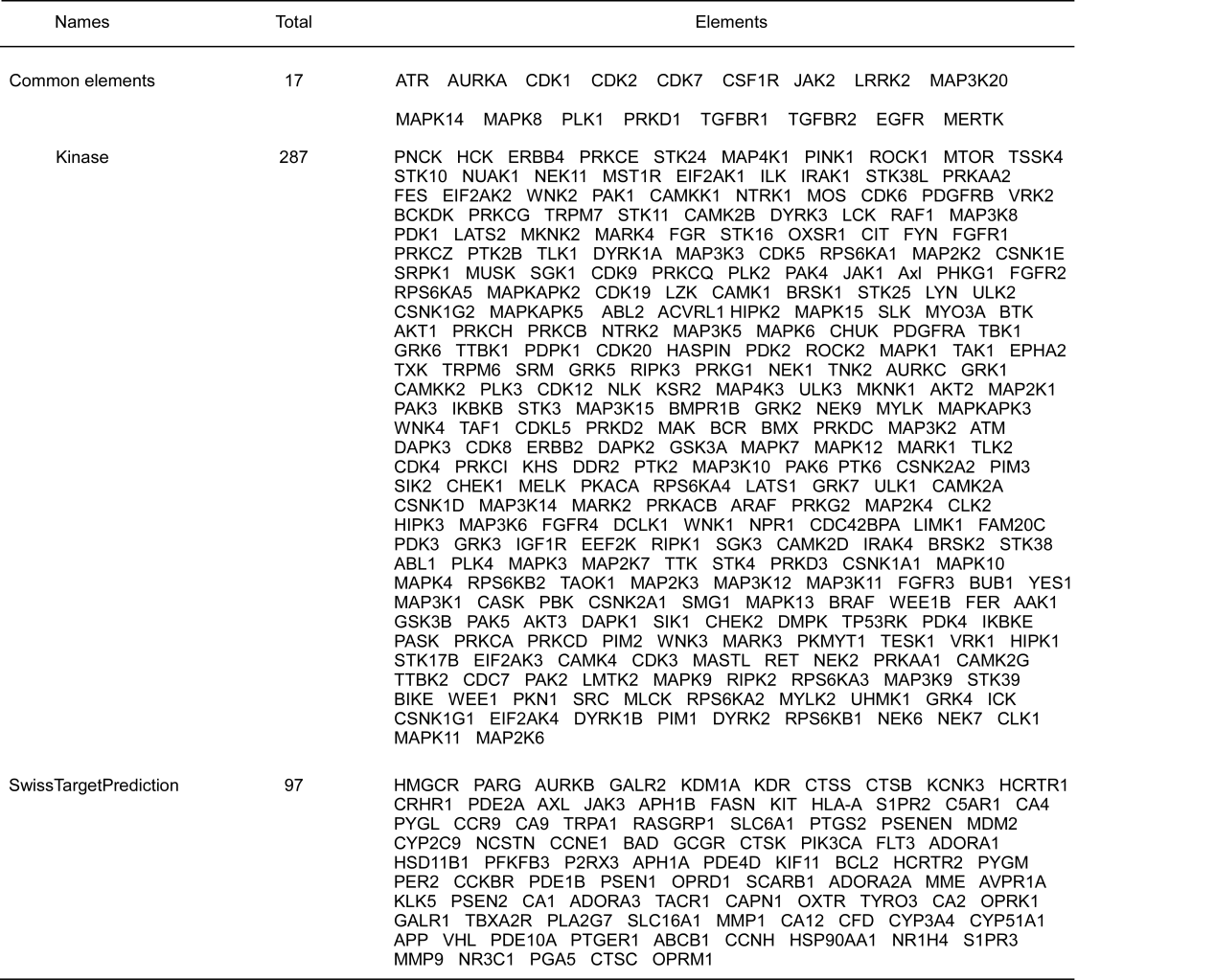


Fig S3. The contents of upstream kinase prediction, SwissTargetPrediction and their intersection.


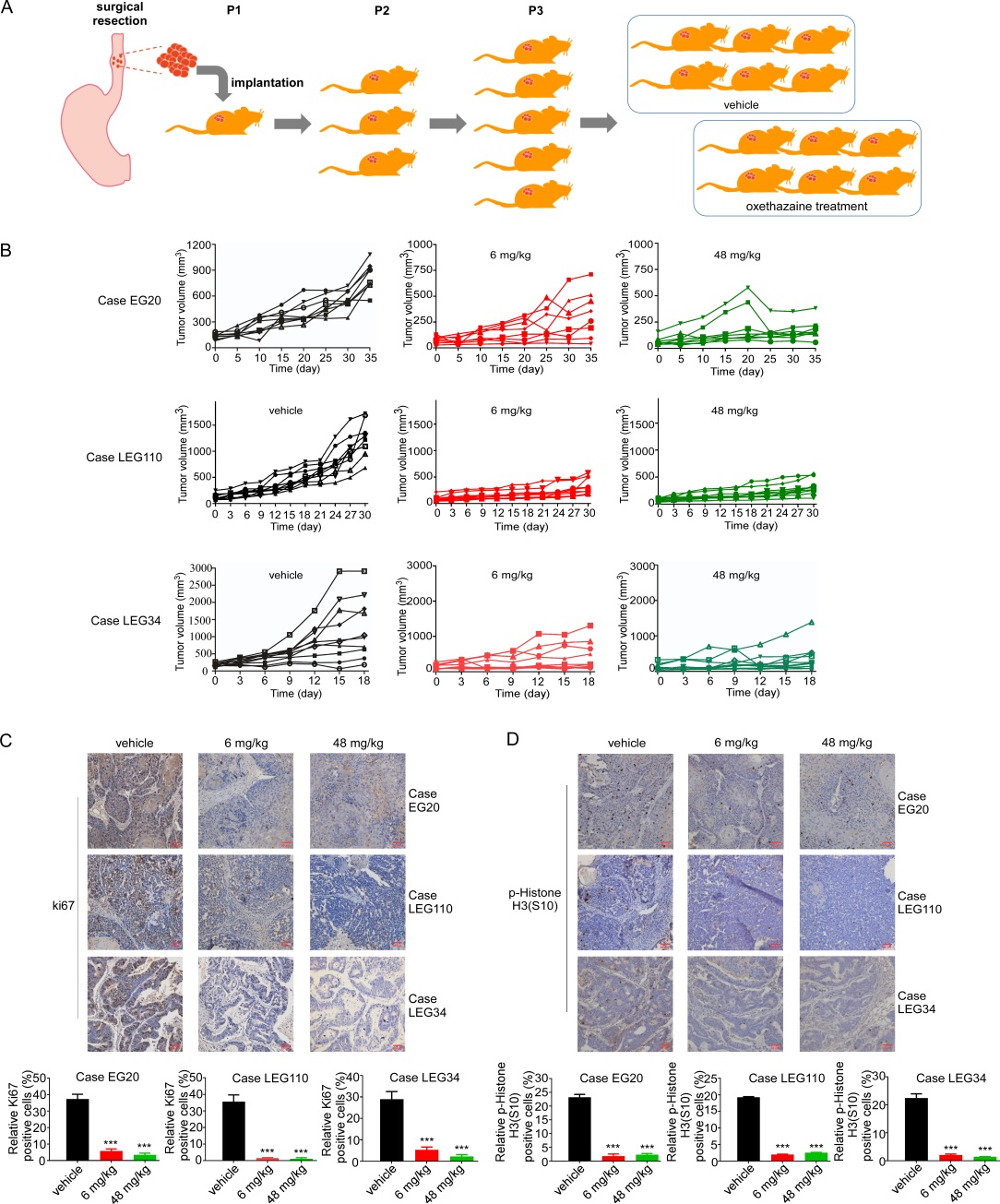


Fig S4. Oxethazaine inhibits ESCC tumor growth and reduce the expresion of Ki67 and p-Histone H3 (S10) *in vivo*. (A) Establishment of PDX models for human esophageal cancer. Patient-derived tumor tissues were cut into small sections and implanted subcutaneously in the backs of immunodeficient mice. The tissues were passaged upon reaching approximately 1000 mm^3^. After stable passaging for 3 generations, the PDX models was established, which were subsequently used for in vivo experiments; (B) The tumor volume changes of each tumor in each PDX model; (C) Immunohistochemistry analysis of Ki67 with DAB staining in the EG20, LEG110, LEG34 PDX cases; (D) Immunohistochemistry analysis of p-Histone H3 (S10) with DAB staining in the EG20, LEG110, LEG34 PDX cases. All data are shown as means±S.D. The asterisks (*, **, ***) indicate statistical significance (*p* < 0.05, *p* < 0.01, *p* < 0.001, respectively).


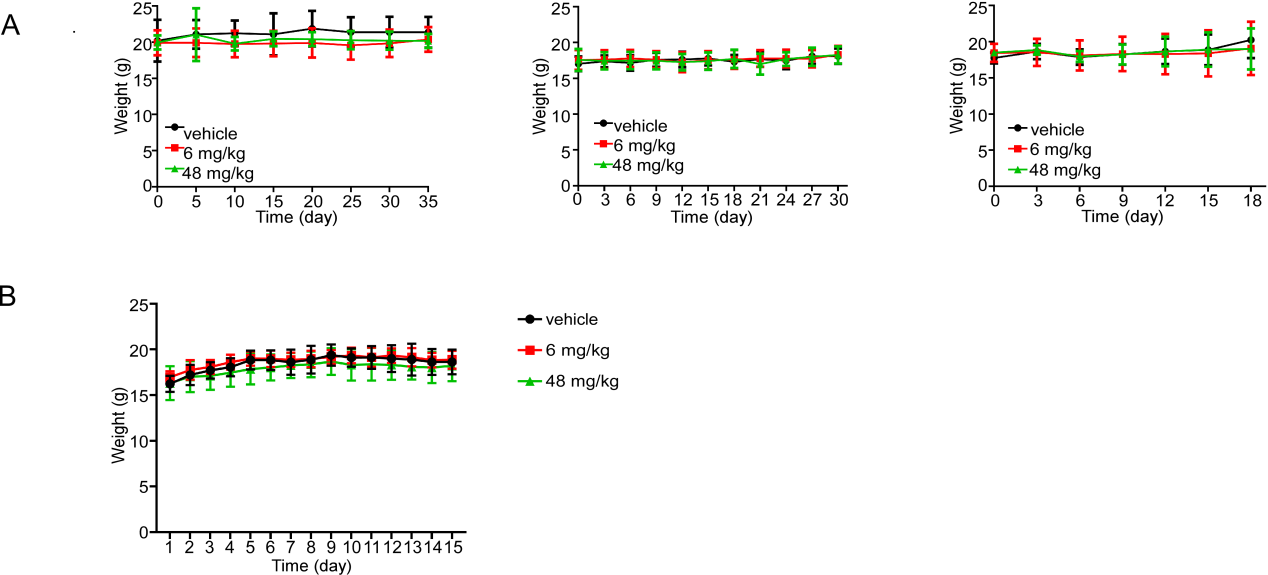


Fig S5. The body weights of the mice were comparable between the vehicle and treatment groups. (A) Body weights of the mice after oxethazaine treated for EG20 (n=8), LEG110 (n=9), LEG34 (n=10). (B) Body weights of the mice after oxethazaine treated in tumor metastasis model. All data are shown as means±S.D.
